# Severe disseminated primary varicella in a patient on long term inhaled corticosteroids

**DOI:** 10.1002/rcr2.1010

**Published:** 2022-07-30

**Authors:** James P. Pham, Greta Beale, Mahdieh Gorji, Benjamin Thompson, Suhel Al‐Soufi, Emily Stone

**Affiliations:** ^1^ Department of Thoracic Medicine and Lung Transplantation St Vincent's Hospital Sydney, School of Clinical Medicine, UNSW Medicine and Health St Vincent's Hospital Darlinghurst New South Wales Australia; ^2^ Dermatology Department Liverpool Hospital Sydney New South Wales Australia; ^3^ Intensive Care Unit St Vincent's Hospital Sydney New South Wales Australia

**Keywords:** asthma, long term inhaled corticosteroids, varicella

## Abstract

Disseminated primary varicella infection can carry risks of significant morbidity and mortality particularly in immunocompromised populations. Routine, funded childhood vaccination against varicella has significantly reduced associated hospitalization and deaths, however, uptake and efficacy among adults is unknown. We present a case of disseminated primary varicella infection (including rash, pneumonitis, hepatitis and thrombocytopenia) in an immunocompetent patient on long term inhaled corticosteroids for asthma. This case highlights potential risk factors for severe varicella which require further study in adults and raises the need to discuss vaccination in at risk groups including appropriate counselling in those who may be at higher risk.

## Graphical abstract

We report a case of severe primary varicella infection with cutaneous, pulmonary and hepatic involvement in an immunocompetent patient, and review the literature for possible risk factors including a recent course of prednisolone and long‐term use of inhaled corticosteroids for asthma. These patients may warrant screening for vaccination against varicella, and counselling as appropriate. Prompt recognition of the characteristic cutaneous and radiographic features of varicella infection can facilitate early diagnosis and initiation of antiviral therapy, conferring better treatment outcomes.

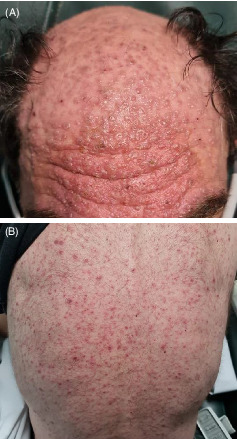

## INTRODUCTION

Disseminated primary varicella infection can carry risks of significant morbidity and mortality, particularly in immunocompromised populations.^1^ Herein, we present a case of disseminated primary varicella infection (including rash, pneumonitis, hepatitis and thrombocytopenia) in an immunocompetent patient with a history of well controlled asthma. Relevant risk factors for severe infection in this case, including a recent course of prednisolone, long term use of inhaled corticosteroids and a history of smoking, are discussed – highlighting the need to screen for vaccination against varicella and counselling as appropriate in such patients who may be at higher risk.

## CASE REPORT

A 42‐year‐old man with a history of asthma presented with a 3‐day history of fevers and a papulovesicular eruption, with new‐onset dry cough. Three weeks earlier, he had been managed for an exacerbation of asthma by his general practitioner a 5‐day course of oral prednisolone (25 mg). His asthma had been treated with inhaled salmeterol and fluticasone (250/25 mcg twice daily) for the preceding 10 years, and he was a former smoker with a 15‐pack‐year history.

**FIGURE 1:**
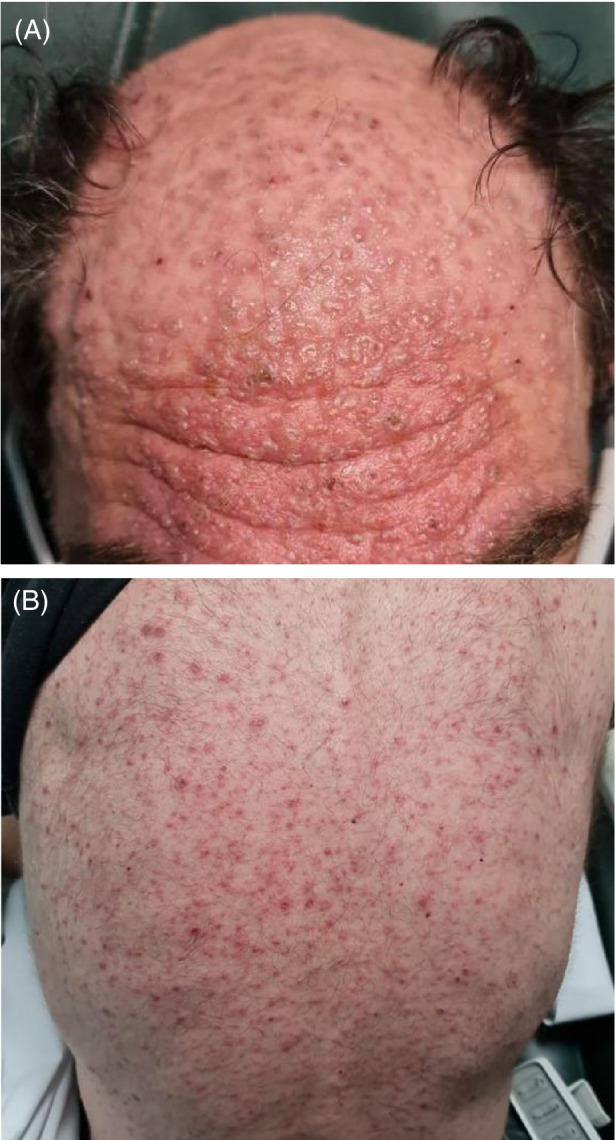
Representative clinical images from presentation showing a polymorphous exanthem characteristic of varicella including (A) umbilicated vesicular eruption involving the face and scalp; and (B) scattered eroded papules on the back. Occasional pustules were also noted on the trunk and forehead.

The rash initially began on the face and rapidly spread in a rostrocaudal fashion to involve his trunk, limbs and genitalia, developing cough on day three. On examination, he was febrile to 39°C but otherwise vitally stable. A polymorphous exanthem was noted with umbilicated vesicles on his face (Figure 1A), eroded papules over his trunk and limbs (Figure 1B) and occasional pustules on his back. Widespread inspiratory crackles and expiratory wheeze were evident on auscultation. A review of systems was otherwise unremarkable.

Arterial blood gas analysis demonstrated Type 1 respiratory failure with primary respiratory alkalosis and adequate metabolic compensation – pO_2_ 55 mmHg (75–100), SpO_2_ 89% (95–100), pCO_2_ 24 mmHg (35–45), HCO_3_^−^ 19 mmol/L (22–28) and pH 7.50 (7.35–7.45). Liver transaminases, neutrophils and C‐reactive protein were elevated, with moderate thrombocytopenia was evident on bloodwork (Table 1). Full blood count, renal function, electrolytes and bilirubin were within reference ranges.

**TABLE 1:** Blood results demonstrating change in biochemical markers from presentation to discharge after antiviral and systemic corticosteroid therapy; with resolving hepatitis, thrombocytopenia and inflammatory markers.

Parameter (units)	Result on presentation (day + 0)	Result on discharge (day + 11)	Reference range
GGT (U/L)	213	149	0–50
ALP (U/L)	267	243	30–110
AST (U/L)	196	44	0–35
ALT (U/L)	235	77	0–40
Albumin (g/L)	21	25	33–48
Plt (×10^9^/L)	80	561	150–400
WCC (×10^9^/L)	10.8	12.8^a^	4.0–10.0
Neutrophils (×10^9^/L)	7.3	10.1^a^	2.0–7.0
CRP (mg/L)	128	67.2	0–5

Abbreviations: ALP, alkaline phosphatase; ALT, alanine aminotransferase; AST, aspartate aminotransferase; CRP, C‐reactive protein; GGT, gamma glutamyltransferase, Hb, haemoglobin; HCO_3_^−^, bicarbonate; pCO_2_, carbon dioxide partial pressure; Plt, platelet count; pO_2_, oxygen partial pressure; SpO_2_, oxygen saturation; WCC, white cell count.

^a^ Elevated neutrophils and white cell count following prednisolone.

**FIGURE 2:**
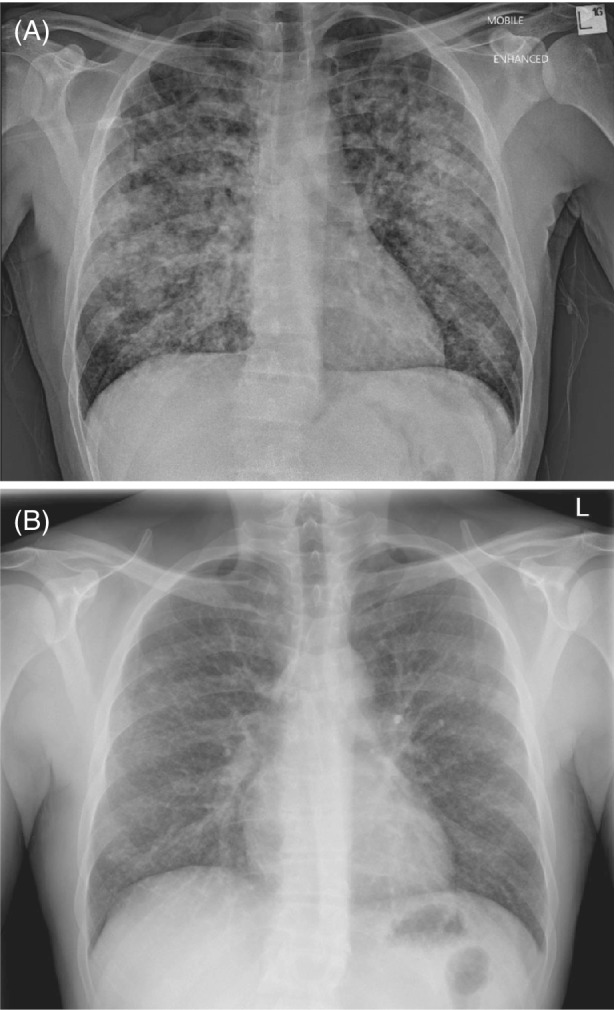
Chest radiographs (A) on presentation showing diffuse nodular air space infiltrates in bilateral lungs, consistent with varicella pneumonitis; and (B) after 1 month showing considerable improvement in micronodular infiltrate which are still apparent.

Chest radiograph featured diffuse micronodular air space infiltrates bilaterally (Figure 2A). Nasopharyngeal viral polymerase chain reaction (PCR) testing (including for SARS‐CoV‐2), sputum culture, urinary legionella and pneumococcal antigens, and mycoplasma serology were negative. Vesicular fluid was positive for varicella zoster virus (VZV) DNA on PCR but negative for herpes simplex viruses 1 and 2, and bacterial culture was negative. The patient denied prior varicella exposure, infection or vaccination, and serology was positive for VZV IgM but negative for IgG – consistent with primary infection rather than reactivation. Screening for primary immunodeficiency (such as lymphocyte or immunoglobulin subsets) was not conducted while the patient was acutely unwell, and he had no social risk factors for secondary immunodeficiency or history of recurrent infections to suggest susceptibility.

He was treated with 5 days of intravenous acyclovir then 5 days of oral valacyclovir, regular salbutamol for wheeze and likely exacerbation of asthma and oral prednisolone for pneumonitis (50 mg then 25 mg, 5 days each). The patient initially required intensive care unit admission for high‐flow nasal prong oxygenation which was gradually weaned to room air; with the vesicular exanthem resolving after 2 weeks. The patient was discharged on day 11 with ongoing clinical and biochemical improvement (Table 1) with follow‐up chest radiograph after 1 month showing marked improvement (Figure 2B).

## DISCUSSION

While varicella is typically associated with mild illness, severe disseminated infections can result in hepatitis, meningoencephalitis, pneumonitis and thrombocytopenia. Immunocompromise is associated with increased mortality rates (up to 7%), although population studies estimate 70%–90% of varicella‐associated deaths occurred in immunocompetent people.^1^ Approximately 90% of varicella infection occurs in children, however, adult age is associated with 23‐to‐29 times higher mortality risk.^1^, ^2^ Pneumonitis and thrombocytopenia may develop in 16% and 30% of adults infected with varicella, respectively, the majority of which are asymptomatic and do not typically cause significant morbidity or mortality in immunocompetent patients.^3^, ^4^

Recent prednisolone use may have predisposed our patient to severe infection – with a case–control study in children demonstrating that systemic steroids in the preceding 30 days increases risk of severe varicella 178‐fold^2^; possibly by impairing interferon‐γ‐mediated T‐cell differentiation. Inhaled corticosteroid (ICS) use in patients with asthma increases risk of bacterial pneumonia,^5^ while a protective effect against influenza pneumonia has been observed.^6^ Long‐term ICS use has been reported in cases of varicella pneumonia^7^, ^8^ and thus may have been a predisposing factor in our patient, however larger controlled studies are required to confirm a causal association. A history of smoking, as in our patient, also increases the risk of severe varicella pneumonia approximately 9‐fold compared to patients who have never smoked.^9^ These patients may therefore benefit from antiviral treatment and close follow‐up upon diagnosis of varicella infection.

Early initiation of intravenous acyclovir correlates with more rapid clinical recovery in acute varicella pneumonia, with use of concurrent systemic corticosteroids associated with earlier improvement of respiratory failure.^10^, ^11^ In Australia, vaccines against VZV been available since 2000 and are recommended for adults without history of natural infection, however they are not funded under the National Immunisations Program for adults. The publicly funded childhood VZV vaccination program in Australia has resulted in a 70% decrease in paediatric varicella hospitalisations since its introduction in 2005.^12^ However, efficacy of varicella vaccination in Australian adults has not been quantified, and thus evidence to support routine or funded vaccines is unclear.

In conclusion, we present a case of severe disseminated varicella in an asthmatic patient on longstanding ICS therapy who had recently received prednisolone. Clinicians caring for similar patients should be aware of this potentially increased risk, and counsel on vaccination as appropriate.

## AUTHOR CONTRIBUTION

All authors were involved in the preparation of the manuscript.

## CONFLICT OF INTEREST

None declared.

## ETHICS STATEMENT

The authors declare that appropriate written informed consent was obtained for the publication of this manuscript and accompanying images.

## Data Availability

Data sharing is not applicable to this article as no new data were created or analyzed in this study.
